# Acquired Zinc Deficiency Mimicking Acrodermatitis Enteropathica in a Breast-Fed Premature Infant

**DOI:** 10.3390/pediatric13030051

**Published:** 2021-08-01

**Authors:** Giovanna D’Amico, Corinne De Laet, Guillaume Smits, Deborah Salik, Guillaume Deprez, Catheline Vilain, Pascale Perlot, Alfredo Vicinanza

**Affiliations:** 1 Department of Pediatrics, Hôpital Universitaire des Enfants Reine Fabiola, Université Libre de Bruxelles, Avenue Jean Joseph Crocq 15, 1020 Brussels, Belgium; pascale.perlot@huderf.be (P.P.); alfredo.vicinanza@huderf.be (A.V.); 2 Nutrition and Metabolic Unit, Hôpital Universitaire des Enfants Reine Fabiola, Université Libre de Bruxelles, Avenue Jean Joseph Crocq 15, 1020 Brussels, Belgium; corinne.delaet@huderf.be; 3 ULB Center of Human Genetics, Department of Genetics, Hôpital Universitaire des Enfants Reine Fabiola, Université Libre de Bruxelles, Avenue Jean Joseph Crocq 15, 1020 Brussels, Belgium; Guillaume.Smits@erasme.ulb.ac.be (G.S.); catheline.vilain@huderf.be (C.V.); 4 ULB Center of Human Genetics, Department of Genetics, Hôpital Erasme, Université Libre de Bruxelles, Route de Lennik 808, 1070 Brussels, Belgium; 5 Interuniversity Institute of Bioinformatics in Brussels, Université Libre de Bruxelles, La Plaine Campus, Triomflaan CP 263, 1050 Brussels, Belgium; 6 Department of Pediatric Dermatology, Hôpital Universitaire des Enfants Reine Fabiola, Université Libre de Bruxelles, Avenue Jean Joseph Crocq 15, 1020 Brussels, Belgium; deborah.salik@huderf.be; 7 Laboratoire Hospitalier Universitaire de Bruxelles (LHUB-ULB), Department of Clinical Chemistry, Université Libre de Bruxelles, Route de Lennik 808, 1070 Brussels, Belgium; guillaume.deprez@lhub-ulb.be; 8 Pediatric Intensive Care Unit, Hôpital Universitaire des Enfants Reine Fabiola, Université Libre de Bruxelles, Avenue Jean Joseph Crocq 15, 1020 Brussels, Belgium

**Keywords:** zinc deficiency, acrodermatitis enteropathica, premature infant, breastfeeding, zinc supplementation

## Abstract

We present a case of a transient acquired zinc deficiency in a breast-fed, 4-month-old-male prematurely born infant, with acrodermatitis enteropathica-like symptoms such as crusted, eroded, erythemato-squamous eruption in periorificial and acral patterns. The laboratory investigations showed low zinc levels in the infant’s and the mother’s serum and in the mother’s milk; genetic analysis did not show any mutation in the *SLC39A4* gene, involved in acrodermatitis enteropathica. Acquired zinc deficiency is often found in premature infants because of their increased requirement, the low serum and milk zinc levels in breastfeeding women being also an important risk factor, as in this case. A prompt zinc supplementation is essential for the good prognosis of the disease.

## 1. Introduction

Zinc is an essential element for the correct growth and development of several tissues, especially of the skin. It has also a key role in the correct functioning of the immune, nervous, and endocrine systems [1,2]. Zinc deficiency may have an inherited or acquired basis [1]. Inherited zinc deficiency, also known as acrodermatitis enteropathica (AE), is an autosomal recessive disorder due to a mutation of the *SLC39A4* gene, which codes for the zinc transporter ZIP4, leading to an impaired absorption of zinc from the gastrointestinal tract.

The acquired types of zinc deficiency could be caused by insufficient intake, such as in cases of low milk zinc levels in breastfeeding women, increased loss, as in gastrointestinal illnesses (recalcitrant diarrhea, intestinal fistulas) or urinary losses (renal diseases), malabsorption, as in case of chronic inflammatory bowel diseases, and increased requirement, such as in preterm babies [1].

In particular, the isolated low zinc levels in a women’s milk could have a genetic basis as well, with a mutation of mother’s *SLC30A2* gene resulting in the dysfunction of the zinc transporter ZnT2, which is responsible for the transfer of zinc into breast milk [1,3]. This pattern leads to the well-defined transient neonatal zinc deficiency (TNZD) [1,3].

The early symptoms are the same in both inherited and acquired diseases including dermatitis, diarrhea and alopecia [1]. However, there are some differences in the onset, in laboratory findings and in the prognosis of these several kinds of zinc deficiency. In all cases, the lack of zinc supplementation could lead to an increased risk of morbidity and mortality in young children. Thus, the prompt identification and treatment of this disorder should be considered as a medical emergency [1,2,3].

## 2. Case Report

**Figure 1:**
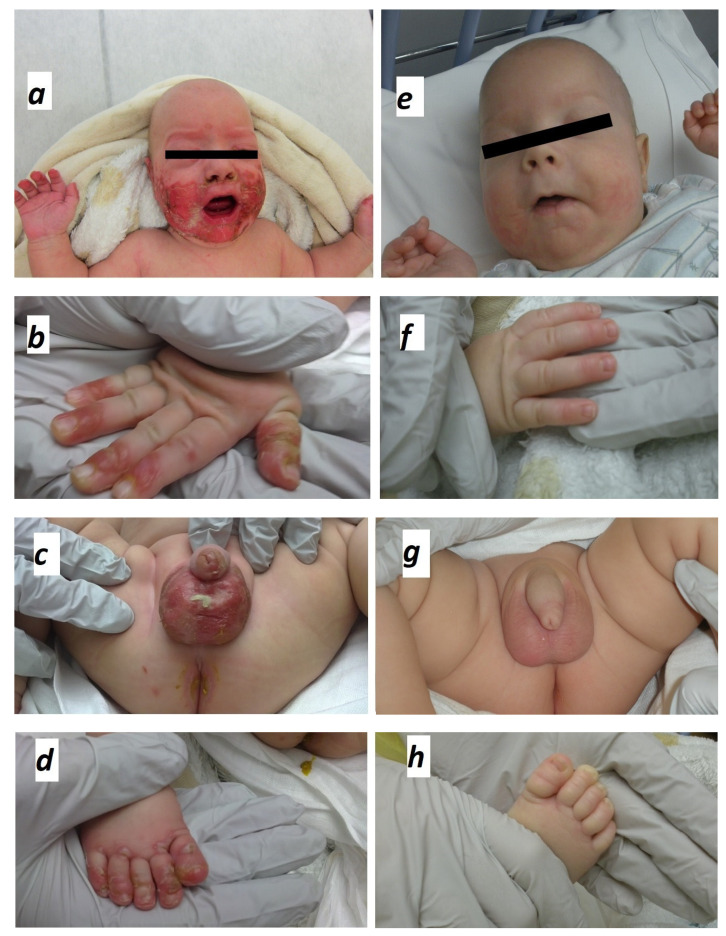
Clinical erosions and typical distribution of skin lesions at the initial presentation of zinc deficiency (panels **a**–**d**) and healing after 2 weeks of zinc supplementation (panels **e**–**h**).

A 4-month-old-male infant presented with eroded, erythemato-squamous eruption characterized by dry, scaly and crusted skin lesions, almost symmetrically distributed in perioral, acral and perineal areas (Figure 1a–d). The fingers and the toes were also involved areas, except for the nails. The patient was born prematurely at 28 weeks (weight at birth: 1200 g) to healthy and unrelated parents in a chorioamnionitis context. Parallel to enteral breast milk feeding, parenteral nutrition was instituted since birth and continued to the 20th day of life. Oligo-elements supplementation, including zinc, was continued until the 34th week of corrected postnatal age (6th week of life) since he was exclusively breast-fed. Statural and weight growths were normal for his corrected age (weight at age of 4 months: 4450 g). At admission, at the age of about 4 months, when the rash first appeared, irritability, diarrhea, and alopecia were associated symptoms.

Impetiginized dermatitis, candidiasis and contact dermatitis were other possible causes. The skin lesions were not typical of candidiasis, fungal infections being excluded by microbiological samples as well. Moreover, the patient’s age and the localization of skin eruptions were not in favor of a contact dermatitis. The impetiginized dermatitis was disproved by the topography of the rash, additional microbiological assessments, and the favorable evolution without antibiotics.

Laboratory and biochemical studies showed decreased zinc levels in the infant’s serum (20 mcg/dL, normal range 67–118 mcg/dL), and maternal serum (68 mcg/dL, normal range 73–137 mcg/dL), as well as in the breast milk (29 mcg/dL compared to the value of 300 mcg/dL of the maternal milk’s control sample). The albumin and alkaline phosphatase (ALP) values in the infant’s serum were normal. The child had methicillin-resistant Sthaphylococcus Aureus (MRSA) and extended spectrum beta-lactamase (ESBL) bacterial colonization of skin lesions. A MRSA decolonization was realized. No other micro-organism was found. Sequencing of the *SLC39A4* gene showed no mutation, excluding classical acrodermatitis enteropathica.

Zinc supplementation was instituted orally at 5 mg/kg/day. The skin lesions improved significantly within three days and resolved completely after 15 days (Figure 1e–h). Long term evolution was favorable after weaning.

## 3. Discussion

We report a case of acquired zinc deficiency affecting an exclusively breast-fed preterm infant of a zinc deficient mother.

The diagnostic suspicion of zinc deficiency was initially supported by the topography of the lesions (several and specific areas involved), the microbiological assessment and the favorable evolution of the rash without antibiotics as well as its immediate improvement after the start of oral treatment with zinc. This was later confirmed by low serum and breast milk zinc levels.

Zinc deficiency may have inherited (as in AE) or acquired causes, some differences helping the differential diagnosis [1,3,4]. In AE, due to a defect of zinc absorption in the gut, the symptoms arise over the weaning whereas in TZND, due to a defect of zinc secretion in the breast milk, the onset of clinical features is at the time of the breast feeding [1,3]. However, TZND is considered as a rare disease; to our knowledge, about fifteen cases have been described since 1985 [5]. Two of the other most common risk factors of zinc deficiency are maternal zinc deficiency and prematurity [3,5,6]. In our case, we found them both.

The zinc level in human milk is higher (>300 mcg/dL) in the first period of breastfeeding and it gradually declines to <100 mcg/dL at 6 months after the delivery [7]. Although the underlying biological mechanism is still unknown, it seems independent from the zinc maternal intake or status and the change occurs in both term and preterm milk [7].

We speculated that the low zinc levels in the breast milk of our patient’s mother might be the consequence of this pattern and more likely of the mother’s serum zinc deficiency as well. However, no maternal *SLC30A2* gene analysis was performed.

Zinc deficiency is more common in premature infants for several reasons. Zinc is accumulated during gestation, so a premature delivery could reduce the zinc tissue stores [8]. Moreover, most of the mother–fetus zinc transfer occurs in the last ten weeks of gestation [2,9]. Several studies also showed that premature infants could have a negative zinc balance until the 60th day of life, probably because the immature gut has a reduced capacity to absorb the zinc so that its excretion appears to be increased [8]. Furthermore, in preterm newborns, zinc requirements might be higher because of the rapid growth and development [8].

In the literature, many reports described patients receiving parenteral nutrition (PN) with associated zinc deficiency [10]. However, patients needing parenteral nutrition, such as preterm infants or patients with non-functioning gastrointestinal tract and consequent malabsorption, tend to already be zinc-depleted, [10]. Moreover, the PN often contains cysteine supplementation to maximize parenteral calcium and phosphate provisions, cysteine may raise urinary zinc losses by increasing the proximal tubular zinc secretion [10].

Our patient received PN until the 20th day of life and oligo-elements supplementation, including zinc, was continued until the 6th week of life. We hypothesized that, even though the patient was supplemented until his 6th week of life, the stocks of zinc were not high enough and, subsequently, exclusive breastfeeding by a zinc-deficient mother probably contributed to the later onset of symptoms. It is noteworthy that zinc deficiency is often seen in case of malnutrition, as the 80% of the zinc is bound to the albumin [11]. However, our patient was not malnourished and had a normal serum albumin level.

As zinc is an essential micronutrient for several functions in the human body, the clinical features of zinc deficiency can be different [1,2]. In particular, the skin is the third most zinc (Zn)-abundant tissue in the body (skeletal muscle 60%, bones 30%, skin 5% and liver 5%). Thus, skin lesions are typical manifestations of the zinc deficiency [1,9]. They consist, as in our case, in papulo-squamous, erosive eruptions involving periorificial, anogenital, and acral areas [12] (Figure 1a–d). As MRSA coexisted with zinc deficiency, it could have worsened the rash. Along with skin lesions, our patient had alopecia and diarrhea as well, thus establishing the classical triad of zinc deficiency [10]. This triad is only observed in 20% to 28% of cases and correlated with the severity of the deficiency [10,13].

Zinc works as a cofactor for polymerases and proteases involved in many functions, such as the intestinal epithelial cell regeneration so that zinc deficiency can cause digestive symptoms as diarrhea [14]. Some manifestations of zinc deficiency are due to its key role in maintaining adequate plasma IGF-I levels, which are essential for cellular proliferation and organism growth [10]. Thus, its suppression can explain symptoms such as growth failure, skin lesions, alopecia and decline in muscle work capacity [10].

We supposed that our patient did not show other manifestations such as muscle weakness or growth failure, because the duration of the deficiency was limited thanks to the quick diagnosis and treatment. The prompt treatment of zinc deficiency is important, especially in infants, because the zinc also plays a key role in neurological development and in immune regulation [1,2,15]. In cases of zinc deficiency, several studies showed reduced humoral and cellular responses with lymphocytes reduction, predominantly T cells and diminished bactericidal and phagocytic capacities of macrophages with an associated immunodeficiency [15].

The treatment consists in zinc supplementation and, in all kinds of zinc deficiencies, approximately 70% of patients have a clinical improvement after six months of treatment, and, frequently, manifestations are already milder after few days [1]. There is not a clear consensus about the dose of zinc supplementation, but elemental zinc at 1–3 mg/kg/day orally should be sufficient [1,13]. The duration of the treatment depends on the etiology. Patients with reversible acquired zinc deficiency could require supplementation for few months in most cases and, generally, there is no relapse after the end of the treatment [1,13]. In contrast, when patients are affected by AE present symptoms after weaning, they usually require life-long supplementation [1,13]. In our case, zinc supplementation was given orally at 5 mg/kg/day. The skin lesions improved significantly after three days and completely healed after 15 days without relapse (Figure 1e–h), suggesting the reversibility of zinc deficiency and a good prognosis.

In conclusion, the aim of this report is to help health workers to recognize, above all by the typical skin lesions, this potentially life-threatening disease, which is rare in developed countries and, therefore, more easily misdiagnosed.

When faced with periorificial, anogenital and acral skin rash, without improvement with antifungal and broad spectrum antibiotics or after excluding skin infections, physicians should think about zinc deficiency. This could be highlighted by analyzing serum and possibly women’s milk zinc levels. In the case of zinc deficiency, a prompt treatment with oral elemental zinc may show immediate improvement of the skin lesions even before laboratory confirmation.

## Data Availability

Not applicable.
